# Supplementary material for: EPSD 2.0: An Updated Database of Protein Phosphorylation Sites Across Eukaryotic Species
Source: Genomics Proteomics Bioinformatics. 2025 Jun 20;23(3):qzaf057. doi: 10.1093/gpbjnl/qzaf057 (PMC12448286; doi:10.1093/gpbjnl/qzaf057)
Supplement: qzaf057_Supplementary_Data [file qzaf057_supplementary_data.zip › supplementary material captions.docx]

**Supplementary material**

**File S1 Supplementary methods**

**Figure S1 Three biological examples of the practical research potential of EPSD 2.0**

**Table S1 A summary of mainstream p-site databases for eukaryotic phosphorylation**

**Table S2 The public data resources that included 10 phosphorylation databases and 100 additional databases or tools**

**Table S3 The distribution of p-sites and phosphoproteins across the 223 species**

**Table S4 The effect types of 88,074 functional events**

**Table S5 The comparison of EPSD 2.0 and EPSD 1.0**
